# Crude triterpenoid saponins from *Anemone flaccida* (*Di Wu*) exert anti-arthritic effects on type II collagen-induced arthritis in rats

**DOI:** 10.1186/s13020-015-0052-y

**Published:** 2015-07-25

**Authors:** Qing Liu, Xiu-Zhen Zhu, Rui-Bing Feng, Zhong Liu, Gui-Yang Wang, Xi-Feng Guan, Guo-min Ou, Yao-Lan Li, Ying Wang, Man-Mei Li, Wen-Cai Ye

**Affiliations:** College of Pharmacy, Jinan University, Guangzhou, 510632 People’s Republic of China; Guangdong Province Key Laboratory of Pharmacodynamic Constituents of TCM and New Drugs Research, Jinan University, Guangzhou, 510632 People’s Republic of China; Guangzhou Jinan Biomedicine Research and Development Center, National Engineering Research Center of Genetic Medicine, Jinan University, Guangzhou, 510632 People’s Republic of China

## Abstract

**Background:**

*Anemone flaccida* Fr . Schmidt (Ranunculaceae) (*Di Wu* in Chinese) is used to treat punch injury and rheumatoid arthritis (RA). However, the active compounds and underlying mechanism of action mediating the anti-arthritic effects of *A. flaccida* remain unclear. This study aims to evaluate the underlying action mechanism of *A. flaccida* crude triterpenoid saponins (AFS) on RA using a type II collagen (CII)-induced arthritis (CIA) rat model, and to assess the anti-inflammatory effects of the main active compounds of AFS, namely flaccidoside II, anhuienoside E, glycoside St-I4a, hemsgiganoside B, hederasaponin B, and 3-*O*-*α*-l-rhamnopyranosyl (1 → 2)-*β*-d-glucopyranosyl oleanolic acid 28-*O*-*β*-d-glucopyranosyl (1 → 6)-*β*-d-glucopyranosyl ester.

**Methods:**

Male Wistar rats (n = 50) were randomly separated into five groups (n = 10) and immunized by CII injection. AFS (200 or 400 mg/kg) and dexamethasone were orally administered for 30 days after establishing the model. The arthritis severity was assessed by paw volume using a plethysmometer. After 30 days of treatment, the right hind paws of the rats were obtained. Paw histology was analyzed by hematoxylin and eosin staining, and radiologic imaging was performed by micro-computed tomography. MTT assays were used to evaluate the cytotoxicity of AFS and its main compounds in RAW264.7 cells. Enzyme-linked immunosorbent assay kits were used to measure interleukin (IL)-6 and tumor necrosis factor (TNF)-α in serum and supernatants from AFS- and main AFS compound-treated RAW264.7 cells stimulated by lipopolysaccharide (LPS).

**Results:**

*Anemone flaccida* crude triterpenoid saponins inhibited redness and swelling of the right hind paw in the CIA model. Radiological and histological examinations indicated that inflammatory responses were reduced by AFS treatment. Moreover, comparing with untreated rats, serum TNF-α (*P* = 0.0035 and *P* < 0.001) and IL-6 (*P* = 0.0058 and *P* = 0.0087) were lower in AFS-treated CIA rats at the dose of 200 and 400 mg/kg/day. AFS and its main compounds, including hederasaponin B, flaccidoside II, and hemsgiganoside B, significantly inhibited TNF-α (*P* = 0.0022, *P* = 0.013, *P* = 0.0015, and *P* = 0.016) and IL-6 (*P* = 0.0175, *P* < 0.001, *P* < 0.001, and *P* < 0.001) production in LPS-treated RAW264.7 cells, respectively.

**Conclusions:**

*Anemone flaccida* crude triterpenoid saponins and its main bioactive components, including hederasaponin B, flaccidoside II, and hemsgiganoside B, decreased pro-inflammatory cytokine levels in a CIA rat model and LPS-induced RAW264.7 cells.

## Background

Rheumatoid arthritis (RA) is a chronic inflammatory autoimmune disease characterized by changes in the synovial tissue. RA is initiated by an infection or tissue injury followed by subsequent inflammatory responses, including joint pain and swelling, as well as synovial hyperplasia, pannus formation, and concomitant destruction of cartilage and bone [1]. Although the exact cause and pathogenesis of RA remain incompletely understood, T-lymphocytes, B-lymphocytes, macrophages, and dendritic cells are involved in the inflammation of the synovial membrane and cartilage–pannus junction [2]. Dysregulated immune cell interactions can induce overproduction of pro-inflammatory cytokines, such as interleukin (IL)-1, IL-6, and tumor necrosis factor (TNF)-α, resulting in an imbalance between pro-inflammatory and anti-inflammatory cytokine activity and joint damage [3].

Despite decades of RA drug development, there remains a lack of viable therapeutic approaches for the prevention and treatment of RA. Steroidal and nonsteroidal anti-inflammatory drugs are approved for alleviation of pain as well as inflammatory and autoimmune components of the disease, without reducing cartilage and bone destruction of joints. However, long-term administration of these drugs can cause severe side effects or toxicity, such as cardiovascular risk and gastrointestinal disturbances [4, 5]. Infliximab has been approved for treatment of RA by blocking TNF-α, but its administration must be considered carefully because of its high cost [6]. Furthermore, antibodies can interfere with immune defense. Thus, there is an urgent need to develop novel therapeutic agents.

The dry rhizomes of *Anemone flaccida* Fr. Schmidt (Ranunculaceae) (*Di Wu* in Chinese) are used to heal fractures and strengthen bones. Previous phytochemical studies demonstrated that triterpenoid saponins are the main chemical components of this plant, as well as the major bioactive constituents [7–9]. Triterpenoid saponins from *A. flaccida* were reported to exhibit anti-inflammatory [10], anticonvulsant [7], anticancer [7], antiviral [11], and immunosuppressive [12] activities. However, the roles of *A. flaccida* triterpenoid saponins for the RA inflammatory response remain unclear.

This study aims to evaluate the underlying mechanism of action of *A. flaccida* crude triterpenoid saponins (AFS) on RA using a type II collagen (CII)-induced arthritis (CIA) rat model, and to assess the anti-inflammatory effects of the main active compounds of AFS, including flaccidoside II, anhuienoside E, glycoside St-I4a, hemsgiganoside B, hederasaponin B, and 3-*O*-*α*-l-rhamnopyranosyl (1 → 2)-*β*-d-glucopyranosyl oleanolic acid 28-*O*-*β*-d-glucopyranosyl (1 → 6)-*β*-d-glucopyranosyl ester, by detecting TNF-α and IL-6 production in lipopolysaccharide (LPS)-treated RAW264.7 cells.

## Methods

### Materials

Dexamethasone was purchased from Guangdong Huanan Pharmaceutical Group Co. Ltd. (China). AFS and dexamethasone were dissolved in distilled water. Bovine CII (2 mg/mL, dissolved in 0.05 M acetic acid), complete Freund’s adjuvant (CFA; 4 mg/mL), LPS (*Escherichia coli* 055:B5), and 3-[4,5-dimethylthiazol-2-yl]-2,5-diphenyltetrazolium bromide (MTT) were purchased from Sigma-Aldrich (USA). TNF-α and IL-6 enzyme-linked immunosorbent assay (ELISA) kits were obtained from eBioscience (USA). Dulbecco’s modified Eagle’s medium (DMEM) and all other cell culture products were acquired from Life Technologies (USA). The murine macrophage cell line RAW264.7 (ATCC No. TIB-71™) was purchased from American Type Culture Collection.

### Preparation of AFS

Rhizomes of *A. flaccida* were collected from Changyang county, Hubei Province, P.R. China, and authenticated by Prof. Guang-Xiong Zhou (Institute of Traditional Chinese Medicine & Natural Products, Jinan University) in accordance with previous reports [9, 13, 14]. A voucher specimen (No. 130512) was deposited in the Institute of Traditional Chinese Medicine and Natural Products, Jinan University, Guangzhou, P.R. China.

Powdered air-dried rhizomes of *A*. *flaccida* (2.6 kg) were extracted with water under reflux three times (first: 20 L, 3 h; second: 15 L, 1 h; third: 10 L, 1 h). The three extracts were combined and concentrated under vacuum to an appropriate volume. The concentrated extract was subjected to a D101 macroporous resin column (50 × 200 cm) and sequentially eluted with H_2_O, 10% ethanol, 30% ethanol, 70% ethanol, and 95% ethanol (v/v). The 70% ethanol solution was collected and concentrated under vacuum to obtain the crude AFS fraction (83 g, dry weight).

### HPLC analysis of AFS

High performance liquid chromatography (HPLC) analysis was performed using an Agilent 1260 system (Agilent Corp., USA) equipped with a quaternary pump, an auto plate-sampler, a thermostatically controlled column apartment, and an evaporative light scattering detector (ELSD; Alltech Corp., USA). Chromatographic separation was carried out on a Cosmosil 5MSII-C_18_ column (250 mm × 4.6 mm, 5 μm) at 30°C with an injection volume of 10 μL. Gradient elution with solvent A (0.2% formic acid in H_2_O) and solvent B (acetonitrile) was performed at a flow rate of 1 mL/min as follows: 20–32% B (10 min); 32% B (15 min); 32–100% B (20 min); 100% B (5 min). AFS was analyzed for six standard saponins, which were previously isolated from the rhizomes of *A. flaccida*. The structures were then identified based on extensive spectroscopic analyses performed in our laboratory. The flaccidoside II content was obtained directly by an external standard method. The contents of the other five saponins (C_x_) were calculated as ratios between the response of each saponin (R_x_) and the response of flaccidoside II in a unit concentration (R_FII_/C_FII_) of the sample solution, using the equation shown below.

$$ {\text{C}}_{\text{x}} = \frac{\text{Rx}}{{{{{\text{R}}_{\text{FII}}} \mathord{\left/{\vphantom {{{\text{R}}_{\text{FII}}} {{\text{C}}_{\text{FII}}}}} \right. \kern-0pt} {{\text{C}}_{\text{FII}}}}}} $$

### Animals

Male Wistar rats (n = 50) [6–8 weeks of age; 120–160 g; SPF grade; Certified No. SCXK (Guangdong) 2011-0015] were purchased from the Department of Laboratory Animal Science at Sun Yat San University (China). All rats were maintained under specific pathogen-free conditions with a standard diet, and provided with water ad libitum. The animals were housed under standard laboratory conditions for 1 week prior to experiments. All experimental procedures complied with the Care and Use of Laboratory Animals, and were approved by the local Animal Ethics Committee of Jinan University.

### Induction of arthritis and drug administration

Experimental arthritis in Wistar rats was induced as described previously [15, 16]. Briefly, 50 animals were arbitrarily divided into five groups (n = 10): normal control; CIA; CIA + AFS (200 mg/kg/day); CIA + AFS (400 mg/kg/day); and CIA + dexamethasone (0.272 mg/kg/day). Bovine CII was dissolved in 0.05 M acetic acid (2.0 mg/mL) and completely emulsified with CFA at a ratio of 1:1. The rats were immunized by intradermal injection of 100 μg CII in CFA into the base of the tail. The rats in the normal control group did not receive an injection. The day of the immunization was defined as day 1. After the immunization with CII, the rats in the AFS treatment groups received an intragastric dose of AFS (200 or 400 mg/kg/day) for 30 days, while the positive control group received dexamethasone (0.272 mg/kg/day). The CIA and normal control groups were administered an equal volume of saline.

Following the immunization, the arthritic condition of each paw, including the severity of joint redness and swelling, was observed and evaluated on alternate days as previously described [15]. Paws were marked with a red line 0.5 cm above the ankle joint, and paw volume was measured three times below the red line using a paw volume plethysmometer. The average of three measurements was considered to be the measured volume. Paw swelling (∆mL) was calculated by subtracting the paw volume on day 1 from the measured volume.

### Histological analysis of paws

The rats were euthanized on day 30 after the CII injection, and the right hind paws of the rats were obtained, stored in 10% neutral formalin, decalcified with 20% ethylenediaminetetraacetic acid for 6 weeks, dehydrated, and embedded in paraffin. Sections were cut along the longitudinal axis, mounted, and stained with hematoxylin and eosin (H&E) [17].

### Radiography

Radiography was performed on the hind paw ankle. The destruction of bone and cartilage was classified and radiologic changes were observed using micro-computed tomography (micro-CT) (ZKKS-MCT-Sharp; Zhongke Kaisheng Medical Technology Co. Ltd., China). The 3D bone architecture was revealed in exquisite detail at 20-μm spatial resolution.

### Determination of serum TNF-α and IL-6

Rats were anesthetized on day 30, and blood was collected from the abdominal aorta and stored at 4°C overnight. The supernatant was collected after centrifugation at 3,000×*g* (Eppendorf 5417R; Eppendorf, Germany) for 10 min. The serum TNF-α and IL-6 levels were measured using specific ELISA kits according to the manufacturer’s recommendations. A standard curve was performed for each plate and used to calculate the absolute concentrations of the indicated cytokines.

### Anti-inflammatory activity of AFS and its main compounds in vitro

#### Cell culture

RAW264.7 murine macrophages were cultured in DMEM supplemented with 10% (v/v) heat-inactivated fetal bovine serum (FBS), 2 mM glutamine, 1 mM sodium pyruvate, 4.5 g/L glucose, 100 μg/mL streptomycin, and 100 U/mL penicillin at 37°C in a humidified atmosphere containing 5% CO_2_.

#### MTT assay

RAW264.7 murine macrophages (5 × 10^3^ cells/well) were cultured in a 96-well plate containing DMEM supplemented with 10% FBS for 24 h until they were nearly confluent. The cells were then treated with AFS (0.055 mg/mL) or AFS-derived compounds (30 μM) for 24 h. The cells were washed twice with phosphate-buffered saline and incubated with 30 μL of MTT (5 mg/mL) for 4 h at 37°C. The medium was discarded, and 100 μL of dimethyl sulfoxide (DMSO) was added. After 15 min of incubation, the absorbances were measured at 570 nm using a microplate reader (Synergy HT; Bio Tek, USA).

#### TNF-α and IL-6 production

To evaluate the anti-inflammatory potential of AFS and its main components, RAW264.7 murine macrophages (5 × 10^5^ cells/well) were cultured in 6-well microplate for 24 h. AFS and its main compounds were initially dissolved in DMSO as stock solutions of 0.22 g/mL and 100 mM, respectively, and then diluted to final concentrations of 0.055 mg/mL and 30 μM in DMEM. Cells were pretreated with AFS and its main compounds for 1 h before stimulation with 100 ng/mL LPS. The activated cells were further incubated for 6 h to measure TNF-α production or 24 h to measure IL-6 production. Supernatants were collected and the concentrations of TNF-α and IL-6 were determined using specific ELISA kits. Control cells were grown under identical conditions, but without the test compounds and LPS. In addition, all compounds were incubated with RAW264.7 cells under identical conditions in the absence of LPS.

### Statistical analysis

Data were presented as mean ± SD from three or more experiments using the Origin 9 statistical package (OriginLab, USA). Student’s *t*-test and one-way analysis of variance (ANOVA) for parametric analysis was used to compare groups and perform multigroup comparisons. The Mann–Whitney *U* test was used to analyze non-parametric data. A *P* value less than 0.05 was considered to be statistically significant, and a *P* value less than 0.01 was considered very statistically significant. Exact *P* values were shown unless *P* < 0.001. Dose-dependency was determined visually by observing the trends of the data.

## Results

### HPLC analysis of AFS

**Fig. 1:**
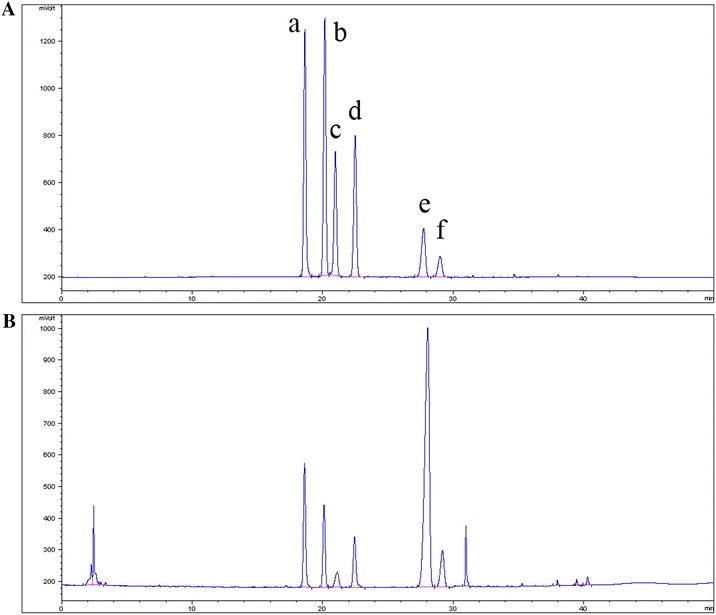
HPLC-ELSD chromatograms of standard saponins (**A**) and AFS (**B**). (*a*) Anhuienoside E. (*b*) Glycoside St-I4a. (*c*) 3-*O*-*α*-l-Rhamnopyranosyl (1 → 2)-*β*-d-glucopyranosyl oleanolic acid 28-*O*-*β*-d-glucopyranosyl (1 → 6)-*β*-d-glucopyranosyl ester. (*d*) Hemsgiganoside B. (*e*) Flaccidoside II. (*f*) Hederasaponin B.

There were significant differences in the quantities of the six main saponins in AFS (Fig. 1). The flaccidoside II content was the highest (28.1%), followed by anhuienoside E (9.5%), glycoside St-I4a (8.9%), hemsgiganoside B (6.2%), hederasaponin B (5.6%), and 3-*O*-*α*-l-rhamnopyranosyl (1 → 2)-*β*-d-glucopyranosyl oleanolic acid 28-*O*-*β*-d-glucopyranosyl (1 → 6)-*β*-d-glucopyranosyl ester (3.9%). The total amount of the six main saponins in AFS was 63.2%.

### Effects of AFS on RA symptoms in CIA rats

**Fig. 2:**
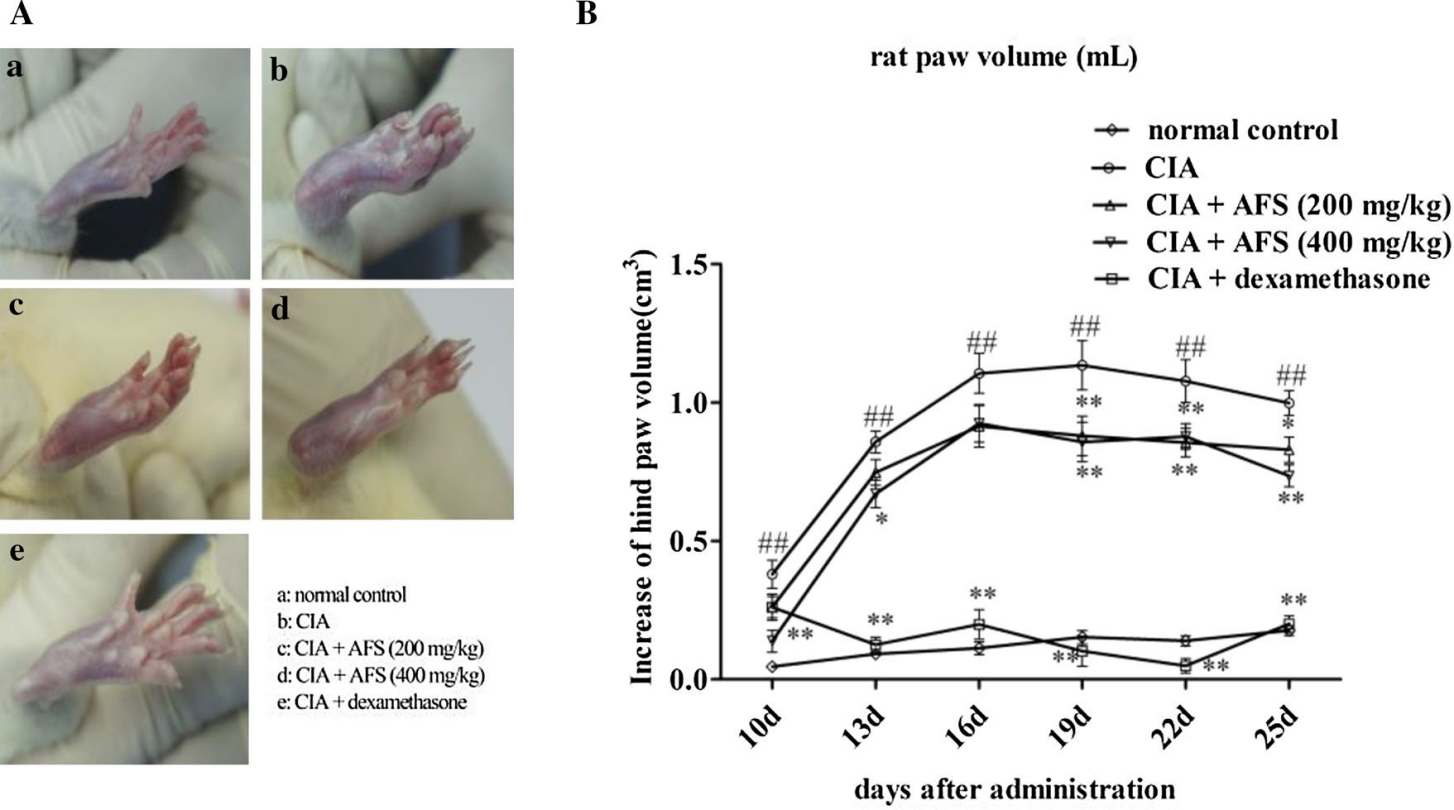
Effects of AFS on paw swelling. **A** Photographs of the hind paws at 30 days after the CII immunization. **B** The severity of arthritis during the course of CIA was determined by measuring the volume of the hind paw using a paw volume plethysmometer. Data were expressed as mean ± SD (n = 10) (^**##**^ *P* < 0.01 CIA group vs. normal control group, *P < 0.05, **P < 0.01 treatment groups vs. CIA group).

Systemic arthritis was induced by injecting CII and CFA on day 1. Onset of arthritis generally occurred at 10 days after the immunization. Clinical manifestations, such as functional impairment and swollen red paws, were observed (Fig. 2A), and the maximal clinical symptoms were achieved at 19 days after the immunization. There was a significant increase in paw volume in the CIA model rats compared with the normal control rats (Fig. 2B; *P* < 0.001). Both doses of AFS (200 and 400 mg/kg) significantly and dose-dependently decreased the paw edema volume and redness compared with the CIA group (*P* < 0.001 and *P* < 0.001, respectively). Dexamethasone (0.272 mg/kg) strongly inhibited arthritis (*P* = 0.0013).

### AFS treatment decreased immune-mediated inflammation and joint damage in CIA rats

**Fig. 3:**
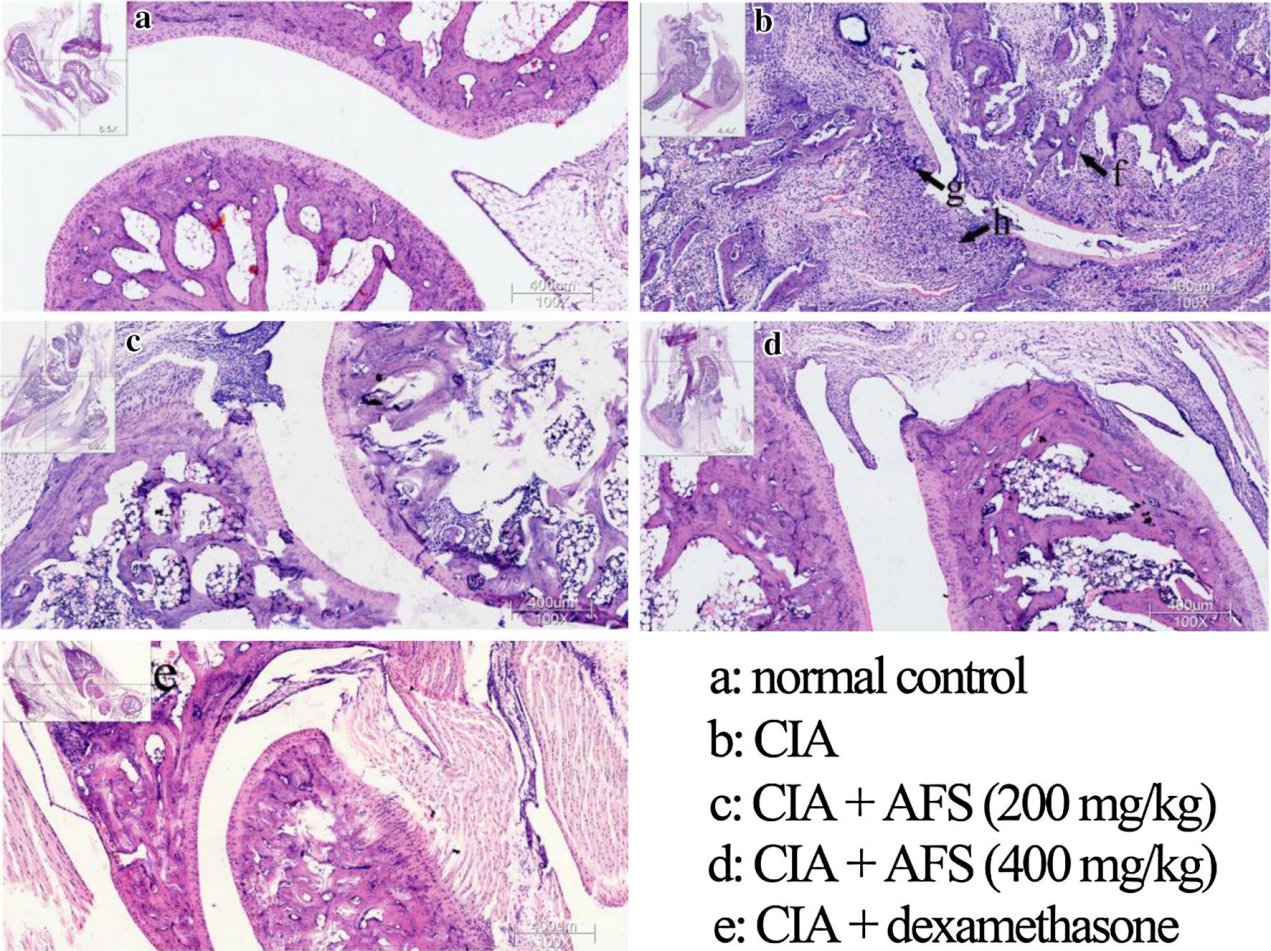
Effects of AFS on histopathological changes. Histological analysis of paws was performed after 30 days of AFS treatment. The upper left images showed histologic staining of ankle joints and the right bottom showed enlarged images of partial area of the upper left images.(*f*) site of bone erosion; (*g*) site of cartilage damage; (*h*) site of synovial inflammation. Original magnification, ×100.

Notable synovial proliferation, granulocyte and mononuclear cell infiltration of the synovial cavity, and partial cartilage and bone destruction were observed in the right hind ankle joints of the CIA rats using H&E staining (Fig. 3a, b). Treatment with 200 or 400 mg/kg AFS remarkably reduced the synovial hyperplasia and inflammatory cell infiltration compared with the CIA rats (Fig. 3c, d). Furthermore, oral administration of dexamethasone significantly reduced the immune cell infiltration, tissue hyperplasia, cartilage damage, and bone erosion compared with the CIA rats (Fig. 3e).

**Fig. 4:**
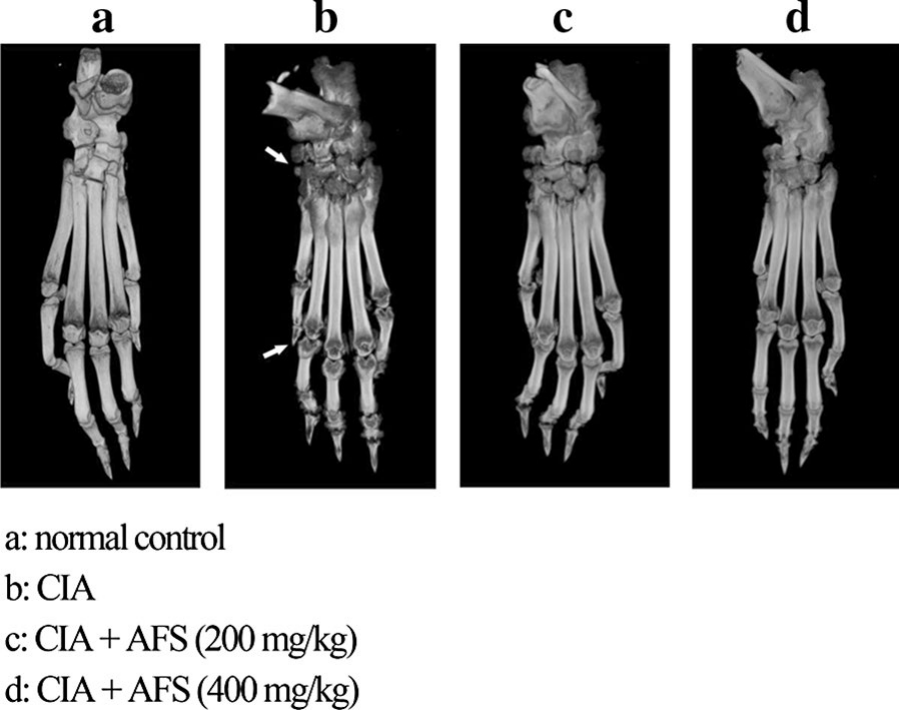
Effects of AFS on joint damage and bone structure in CIA. Representative radiographs obtained after 30 days of AFS treatment demonstrating remarkable amelioration of the articular destruction. *Arrows* indicated remarkable soft tissue swelling and large bone erosion.

Radiography was performed on the hind paws after euthanasia. Radiological changes, such as soft tissue swelling and bone erosion, were observed in the CIA rats (Fig. 4a, b). In contrast, the rats treated with AFS showed a significant reduction in soft tissue swelling and cartilage and bone destruction. Joint space enlargement and thickening of the periosteum were ameliorated after AFS treatment (Fig. 4c, d).

### AFS decreased TNF-α and IL-6 levels in serum

**Fig. 5:**
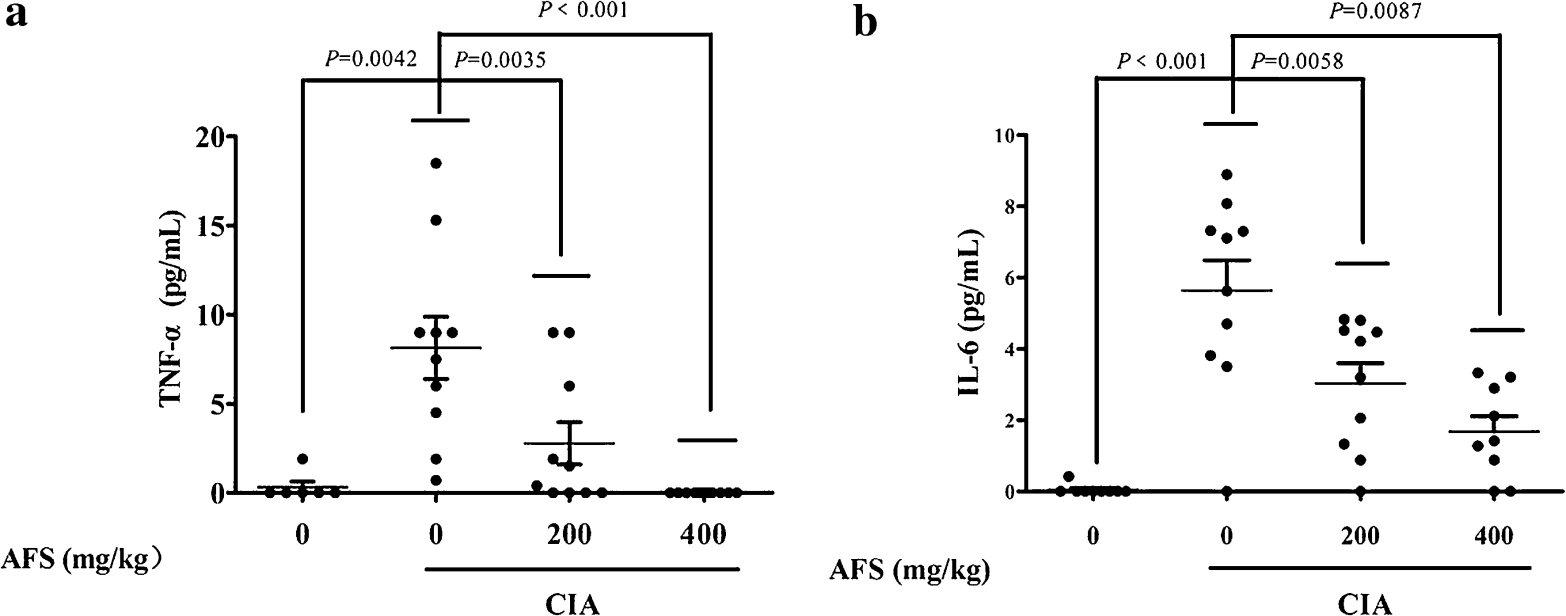
Effects of AFS on cytokine production in rat serum. **a** The concentrations of TNF-α and **b** IL-6 in serum collected from CIA rats treated with AFS. Data were expressed as means ± SD (n = 10).

Low levels of TNF-α and IL-6 were observed in the serum of the normal control rats (Fig. 5). However, these cytokines were significantly increased in the serum of the CIA rats (*P* = 0.0042 and *P* < 0.001, respectively). Treatment with AFS at 200 mg/kg (*P* = 0.0035 and *P* = 0.0058) and 400 mg/kg (*P* < 0.001 and *P* = 0.0087) markedly inhibited the production of TNF-α and IL-6, respectively, in the serum of the CIA rats.

### AFS and its main components suppressed TNF-α and IL-6 production in LPS-treated murine macrophages

**Fig. 6:**
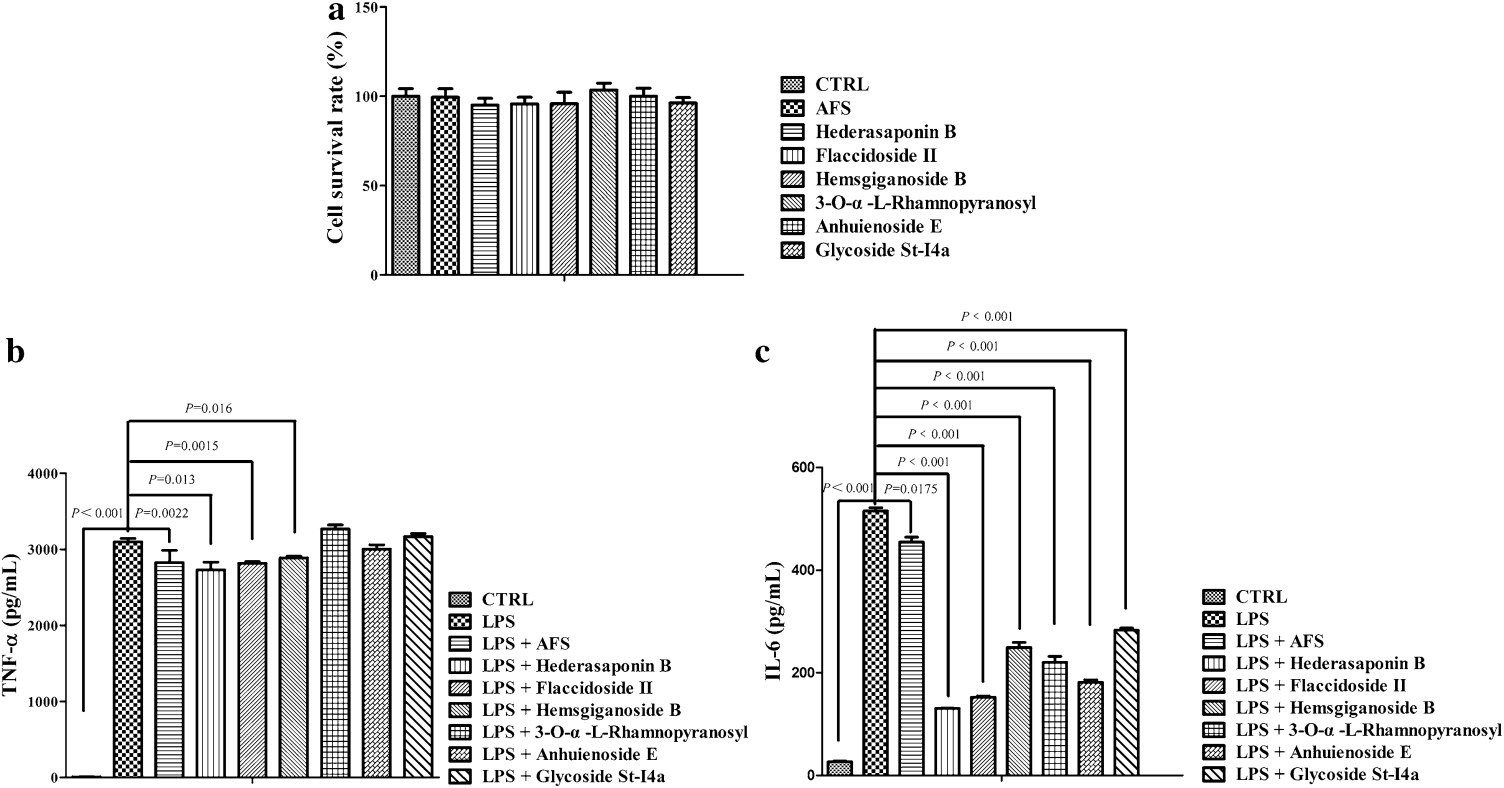
Effects of AFS and its components on LPS-induced TNF-α and IL-6 production in macrophages. **a** Cytotoxicity in RAW264.7 cells treated with AFS and its main components. RAW264.7 murine macrophages were cultured in a 96-well plate for 24 h, and then incubated with or without AFS (0.055 mg/mL) or its main components (30 μM) for 24 h. Cell proliferation was determined by the MTT assay. **b**–**c** Inhibitory effects of AFS components on LPS-induced TNF-α and IL-6 release in macrophages. Cells were incubated with AFS (0.055 mg/mL) or its main components (30 μM) for 1 h, followed by stimulation with or without LPS (100 ng/mL). The production of TNF-α and IL-6 in culture supernatants was measured by ELISA. Data were presented as mean ± SD of three independent experiments.

RAW264.7 cells were pretreated with AFS and the individual compounds such as hederasaponin B, flaccidoside II, hemsgiganoside B, 3-*O*-*α*-l-rhamnopyranosyl (1 → 2)-*β*-d-glucopyranosyl oleanolic acid 28-*O*-*β*-d-glucopyranosyl (1 → 6)-*β*-d-glucopyranosyl ester, anhuienoside E, and glycoside St-I4a at concentrations of 0.055 mg/mL and 30 μM, respectively, which did not induce cytotoxicity (*P* = 0.178, *P* = 0.263, *P* = 0.117, *P* = 0.488, *P* = 0.157, *P* = 0.345, and *P* = 0.407), followed by stimulation with 100 ng/mL LPS to investigate the effects of AFS and its main components on LPS-induced TNF-α and IL-6 production (Fig. 6a). Pretreatment with 3-*O*-*α*-l-rhamnopyranosyl (1 → 2)-*β*-d-glucopyranosyl oleanolic acid 28-*O*-*β*-d-glucopyranosyl (1 → 6)-*β*-d-glucopyranosyl ester, anhuienoside E, and glycoside St-I4a significantly inhibited IL-6 production in LPS-treated macrophages (Fig. 6c; *P* < 0.001, *P* < 0.001, and *P* < 0.001, respectively). Furthermore, AFS, hederasaponin B, flaccidoside II, and hemsgiganoside B, significantly inhibited both TNF-α (Fig. 6b; *P* = 0.0022, *P* = 0.013, *P* = 0.0015, and *P* = 0.016, respectively) and IL-6 (Fig. 6c; *P* = 0.0175, *P* < 0.001, *P* < 0.001, and *P* < 0.001, respectively) production in LPS-treated macrophages.

## Discussion

In the present study, oral administration of AFS from *A. flaccida* significantly inhibited joint swelling, synovial hyperplasia, and inflammatory cell infiltration, thereby alleviating joint damage in CIA rats. Furthermore, AFS remarkably reduced the TNF-α and IL-6 levels in the serum of these rats. Triterpenoid saponins were the major active components of AFS, and most of these components as well as AFS inhibited TNF-α and IL-6 production in LPS-stimulated RAW264.7 macrophages.

The phytochemical and pharmacological properties of *A. flaccida* have been studied extensively. Triterpenoid saponins may contribute to the anti-inflammatory and immunosuppressive activities of *A. flaccida* both in vivo and in vitro [12]. Our chemical analysis revealed that AFS contained six major triterpenoid saponins, namely flaccidoside II, anhuienoside E, glycoside St-I4a, hemsgiganoside B, hederasaponin B, and 3-*O*-*α*-l-rhamnopyranosyl (1 → 2)-*β*-d-glucopyranosyl oleanolic acid 28-*O*-*β*-d-glucopyranosyl (1 → 6)-*β*-d-glucopyranosyl ester. Of these, flaccidoside II and glycoside St-I4a inhibited LPS-stimulated overexpression of cyclooxygenase-2 and prostaglandin E2, which are involved in inflammatory diseases [7]. Hederasaponin B suppressed *N*-formyl-methionyl-leucyl-phenylalanine-induced superoxide generation in human neutrophils [18]. In the present study, the six main saponins in AFS possessed anti-inflammatory activity, through the suppression of TNF-α and IL-6 production in LPS-stimulated RAW264.7 macrophages. Moreover, the AFS used in the present study contained 63.2% triterpenoid saponins, including flaccidoside II (28.1%), glycoside St-I4a (8.9%), and hederasaponin B (5.6%).

The AFS-administered rats showed marked reductions in paw volume compared with the CIA model rats. Both synovial tissue damage and bone destruction in the joint are hallmarks of RA caused by thickening of the synovial lining and the formation of invasive pannus tissue [3, 19]. Synovial hyperplasia and inflammatory cell infiltration in the joint were significantly decreased in the AFS-treated rats compared with the CIA rats, and these effects were confirmed by the results of micro-CT examination.

Although published data regarding the mechanism by which *A. flaccida* prevents RA are limited, pro-inflammatory cytokines and mediators are involved in the pathogenesis of arthritis [3, 20]. Among these, TNF-α is one of the most important cytokines involved in RA, causing synovial inflammation and hyperplasia, and thereby facilitating the degradation of articular cartilage and bone [21, 22]. A TNF-α-specific antibody attenuated the symptoms of spontaneously developed arthritis in human TNF-α transgenic mice [5]. Prophylactic administration of soluble chimeric TNF-α receptors reduced the incidence and severity of RA in a mouse model [23]. These findings might be related to the ability of TNF-α inhibitors to induce inflammatory cell apoptosis, exert direct cytotoxicity, and alter the influx and efflux of inflammatory cells in joints [24]. Moreover, Romano et al. [25] revealed that TNF-α inhibitors induced synoviocyte apoptosis by modulating the Fas/FasL death receptor pathway. IL-6 is a soluble cytokine that regulates local inflammation and immune responses [26]. In RA, IL-6 is abundantly expressed in the synovial tissue, and causes bone destruction and the development of osteoporosis by promoting synoviocyte proliferation and osteoclast differentiation [27, 28]. IL-6 also enhances angiogenesis and increases vascular permeability of synovial tissue by stimulating excess production of vascular endothelial growth factor. In addition, IL-6 promoted Th17 differentiation and inhibited transforming growth factor-β-induced regulatory T cell differentiation, thereby disrupting immunological tolerance [29, 30]. The serum TNF-α and IL-6 levels were markedly decreased in the AFS-treated rats, and the main components of AFS significantly inhibited LPS-induced TNF-α and IL-6 production in vitro. These results suggest that AFS improved the symptoms and signs of RA, probably through inhibition of TNF-α and IL-6 production.

## Conclusions

*Anemone flaccida* crude triterpenoid saponins and its main bioactive components such as hederasaponin B, flaccidoside II, and hemsgiganoside B decreased pro-inflammatory cytokine levels in a CIA rat model and LPS-induced RAW264.7 cells.

## Abbreviations

AFS: *Anemone flaccida* crude triterpenoid saponins
ATCC: American Type Culture Collection
CII: type II collagen
CIA: type II collagen induced-arthritis
CFA: complete Freund’s adjuvant
DMEM: Dulbecco’s modified Eagle’s medium
DMSO: dimethyl sulfoxide
ELISA: enzyme-linked immunosorbent assay
ELSD: evaporative light scattering detector
FBS: fetal bovine serum
HPLC: high performance liquid chromatography
IL-6: interleukin-6
IL-1: interleukin-1
LPS: lipopolysaccharide
MTT: 3-[4,5-dimethylthiazol-2-yl]-2,5-diphenyltetrazolium bromide
micro-CT: micro-computed tomography
RA: rheumatoid arthritis
SD: standard deviation
TNF-α: tumor necrosis factor-alpha
